# Supplementary material for: Aggregation state of Mycobacterium tuberculosis impacts host immunity and augments pulmonary disease pathology
Source: Commun Biol. 2021 Nov 3;4:1256. doi: 10.1038/s42003-021-02769-9 (PMC8566596; doi:10.1038/s42003-021-02769-9)
Supplement: Supplementary file 2 — Supplementary Information [file 42003_2021_2769_MOESM2_ESM.pdf]

Aggregation state of *Mycobacterium tuberculosis* impacts host immunity and augments pulmonary disease pathology

Afsal Kolloli[1], Ranjeet Kumar[1], Pooja Singh[1,2], Anshika Narang[1], Gilla Kaplan[3], Alex Sigal[4,5,6], Selvakumar Subbian[1,*]

Supplementary Table-1. Description of primers used in qPCR

Gene	Sequence (5'-3')
NLRP3	GCCACTGTGATATGCCATGA
	CACATGCTTGCGGTACTTCT
PPARG	GGCGCGTATCTTGAAGAACT
	GTTCGAGCCACAGGAATCAT
CD14	GCTATGCTGACGTAGTCAAG
	GGTGCCAGTTACCTCTATGT
CRP	GCCAGAGGCAAGCATTATTC
	GCAGGCACACGCAATGATGG
IL4	CCATGCACCAAGCTGATCAT
	TCCTTGAAGCACCAAGACAC
MMP1	AATGGCTAAGGAAGGCCAAG
	ATCAGGATGATGCGAGTGAC
MMP9	CGCCAGCTACGACAAGGACA
	AAGTGGTGGCACACCAGAGG
ARG1	GGCATCTACATCACAGAAGC
	CTGTGTTCACCGTTCGAGTT
CAP18	GATGCCTTCAACCAGCAGTC
	CTCCGTCTCCTTCACCGTAA
HIF1A	CCTCACCAAACACAGCAGGA
	TTCCGCTTTCTCTGGGCATT
S100A8	AATTACCACGCCCTCTACGG
	CGGACACGCCTATCTTCACA
S100A9	AGACATCTGTGGGCTCCTCT
	GGTATCGATGCTGCGTTCCA
S100A12	GATCACCACGGAACTCGTGA
	CACTCAGAGCGCTTGCTACT
TBX21	CCTCGCTCTGGACTGACATC
	CCACTGGAAGGATAGGGGGA
PRF1	AACAATCCCAGGTGGACGAC
	GTCATCCCAGCCATAGTCCG
KLRG1	ACTGGCTGCTGGCATAATGT
	AATCACCGATCCCCAGAAGC

SUPPLEMENTARY FIGURE-1

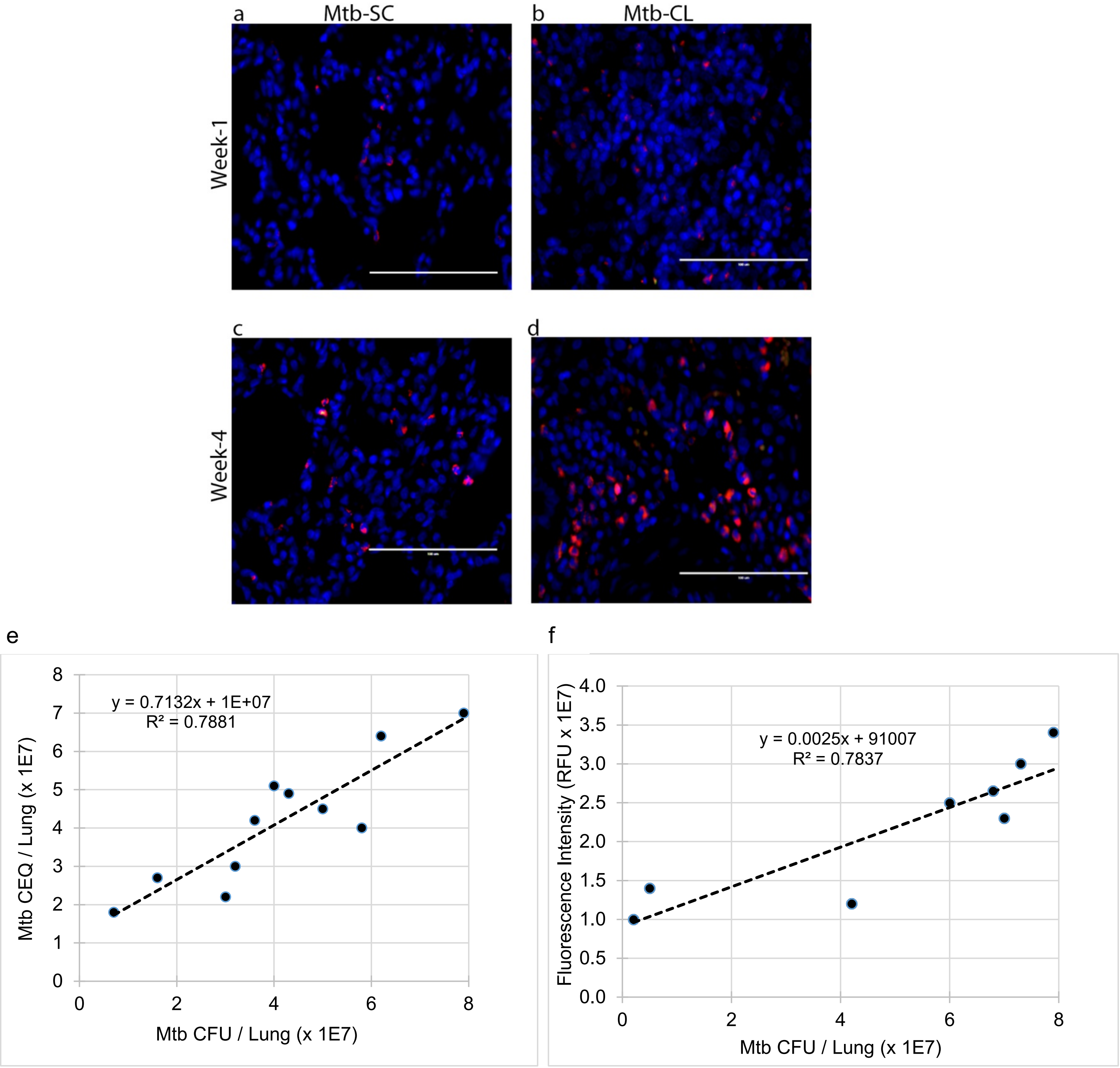

Supplementary Figure-1. Fluorescent imaging of Mtb-SC and Mtb-AG in rabbit lungs at one week (a and b) or 4 week (c and d) post infection. A positive correlation between Mtb CFU and CEQ was noted in rabbit lungs at 4 weeks post-infection (e), and a positive correlation was observed between Mtb CFU and bacterial fluorescence intensity measured in rabbit lungs at 4 weeks post-infection (f).

SUPPLEMENTARY FIGURE-2

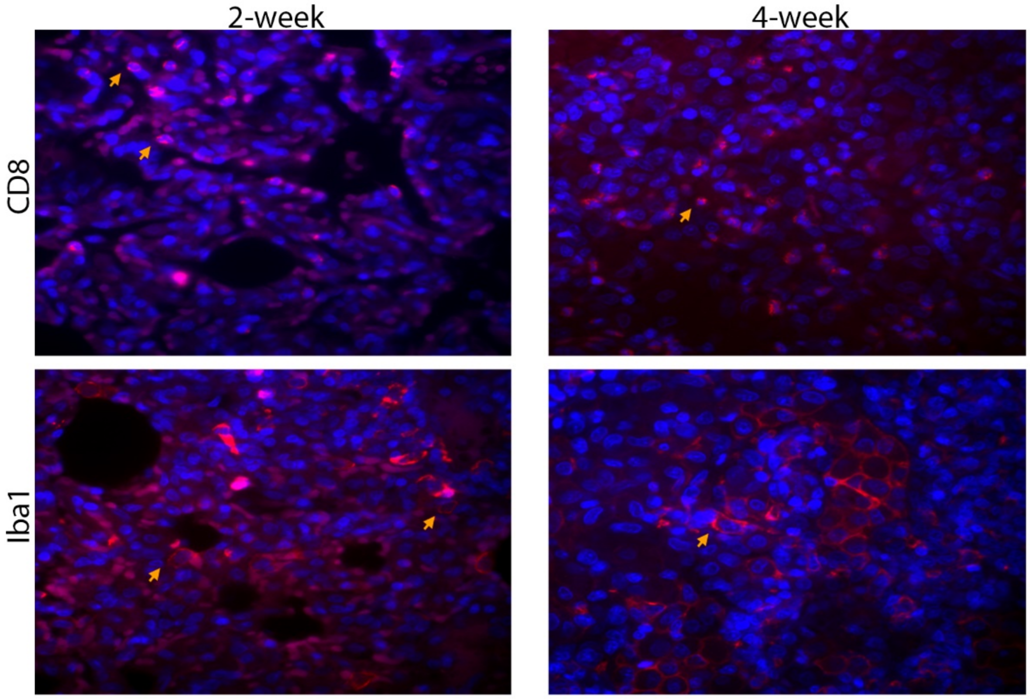

Supplementary Figure-2. Representative immunofluorescent imaging showing CD8+ and IBA1+ (macrophages) in rabbit lung sections (red/pink spots; yellow arrows) infected with Mtb-AG or Mtb-SC at 2 and 4 weeks post-infection. The nucleus is stained with DAPI (blue).

SUPPLEMENTARY FIGURE-3

a. Summary of Rabbit lung RNAseq experiment

Sample	Replicates	Number of input reads	Uniquely mapped reads (number)	Uniquely mapped reads (%)
Uninfected	N_R1	36282072	23909112	65.9
	N_R2	28198175	18890941	66.99
	N_R3	30614025	20213419	66.03
SC-infection	SC_R1	25433041	18342312	72.12
	SC_R2	30361169	22712991	74.81
	SC_R3	27830389	20931980	75.21
AG-infection	AG_R1	23835217	18235694	76.51
	AG_R2	29299748	21645956	73.88
	AG_R3	28403800	20131424	70.88

b. Alignment plot (number of reads)

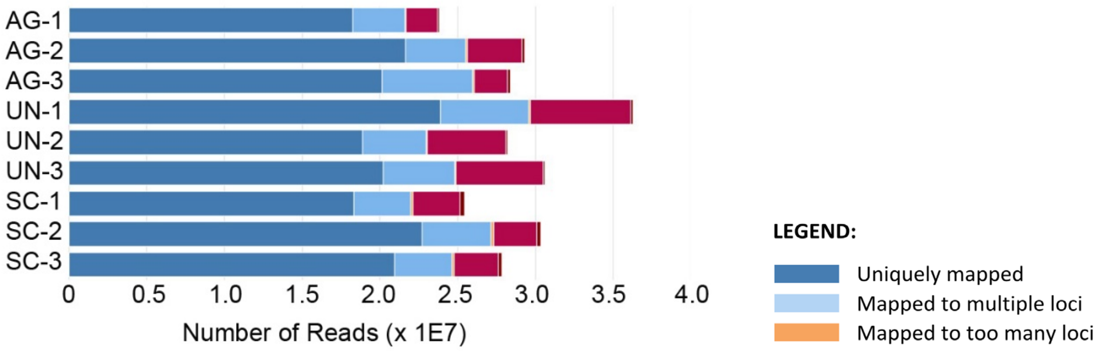

c. Alignment plot (percentage of reads)

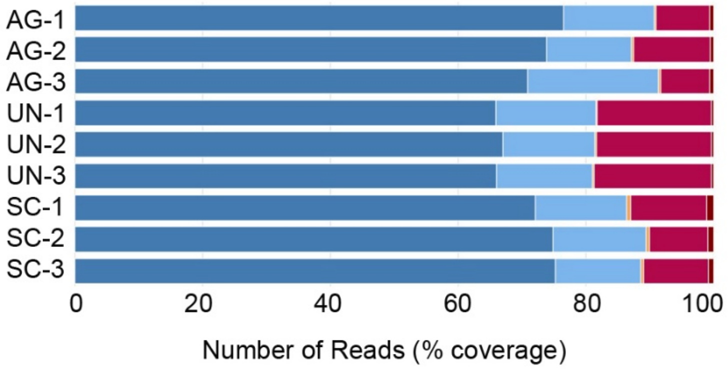

Supplementary Figure-3. Summary of RNAseq data from uninfected or Mtb-AG or Mtb-SC infected rabbit lungs at 24 hours post-infection. a. The sample type and replicates of sample used and the number of input and mapped reads and percentage of uniquely mapped reads. b. Alignment plot of RNAseq represented as the number of reads (x-axis) and sample type with replicates (y-axis). c. Alignment plot of RNAseq represented as percentage of reads (x-axis) and sample type with replicates (y-axis). The experiment was performed in triplicates for each sample type. The legend is common for both b and c.

SUPPLEMENTARY FIGURE-4

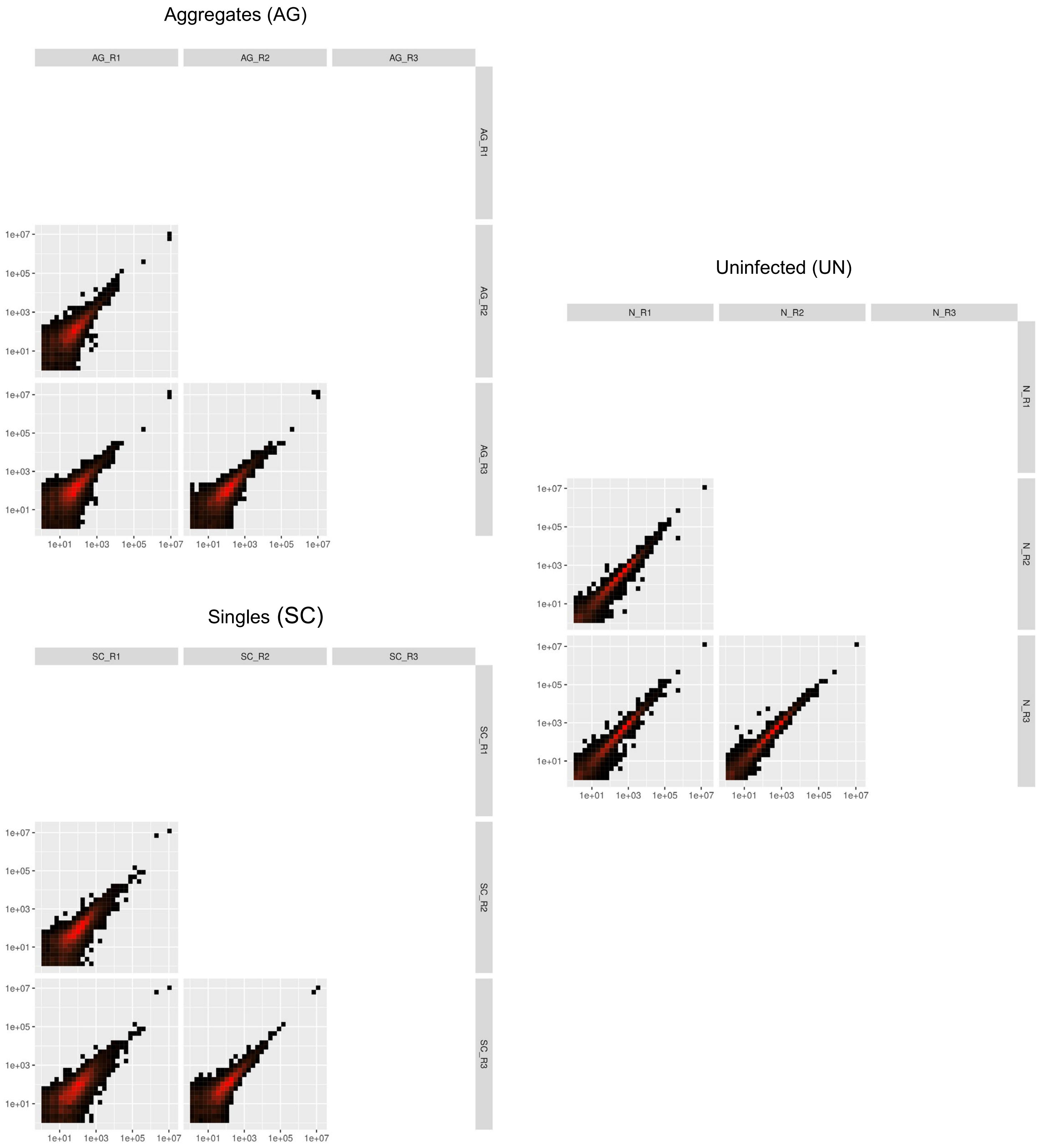

Supplementary Figure-4. Reproducibility of data among biological replicates used in RNAseq experiments. The plot shows significant consistency among the RNAseq reads obtained from triplicates of Mtb-AG or Mtb-SC infected or uninfected (UN) rabbit lung samples.

SUPPLEMENTARY FIGURE-5

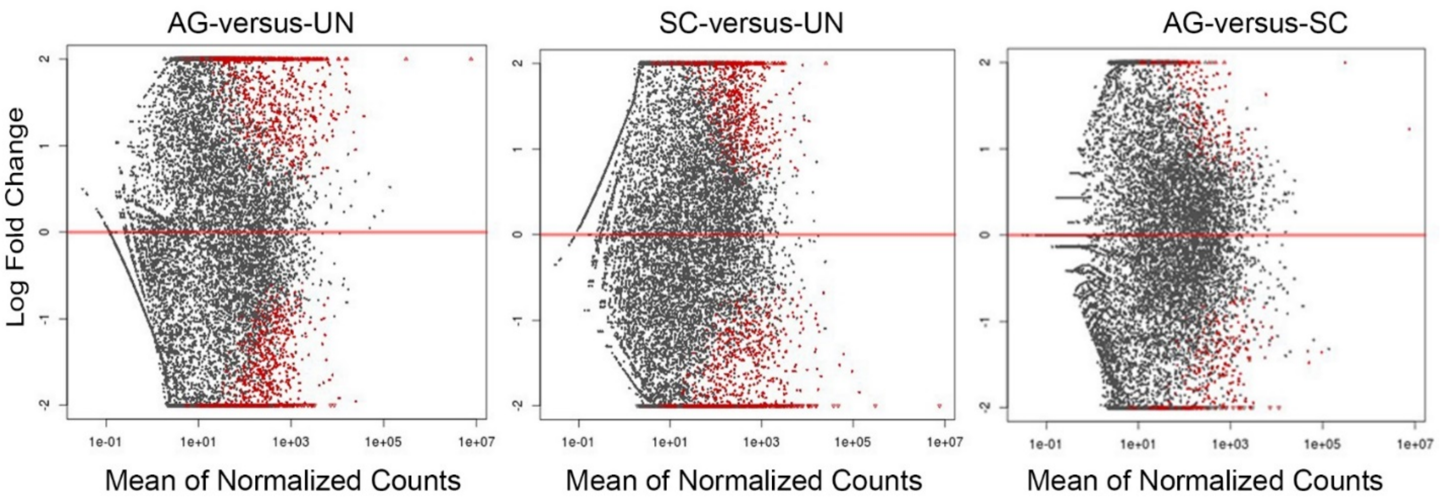

Supplementary Figure-5. MA plot showing the distribution of mean of normalized gene counts in Mtb-AG versus uninfected (UN), Mtb-SC versus UN and Mtb-AG versus Mtb-SC data. Each dot is a gene count and y-axis shows the fold change in gene expression in log scale. Dots in red color are significantly differentially expressed in each of the comparator groups.

SUPPLEMENTARY FIGURE-6

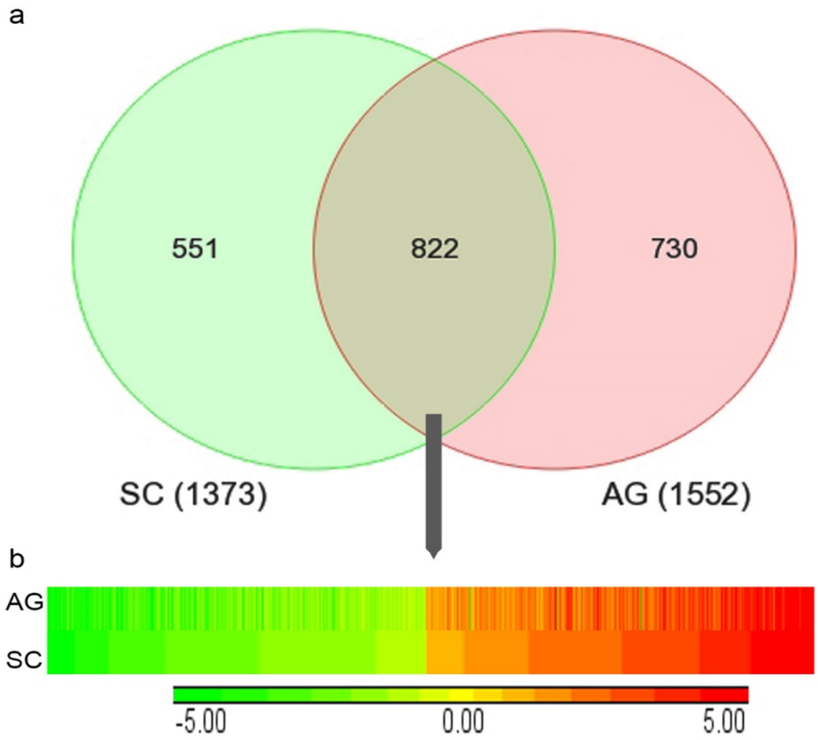

Supplementary Figure-6. RNAseq analysis of host gene expression in rabbit lungs during Mtb-AG and Mtb-SC infection. a. Venn diagram showing significantly differentially expressed genes (SDEG) in Mtb-SC or –AG infected rabbit lungs at 24 hours (n=3 animals/group). Uninfected samples were used for normalization of gene expression from Mtb-infected samples. b. Heat map of SDEGs commonly perturbed during both Mtb-SC and -AG infection. Red color represents upregulation and green color represents downregulation of gene expression. The scale bar ranges from -5 (green) to +5 (red).

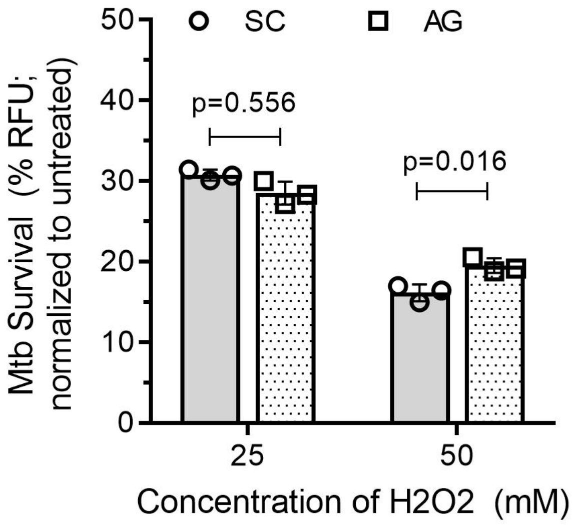

Supplementary Figure-7. Mtb-AG is more tolerant than Mtb-SC to exposure to hydrogen peroxide, an ROS generating agent. Broth cultures of fluorescent Mtb AG and Mtb SC expressing mCherry were exposed to hydrogen peroxide at 25 or 50 mM, and bacterial viability was measured after 72 hours as fluorescent units. Untreated Mtb cultures were used to normalize the data from treated cultures. The experiment was repeated three times in duplicates. Values plotted are mean +/- standard error. Data were analyzed by Mann-Whitney U-test. RFU-Relative Fluorescence Unit.

SUPPLEMENTARY FIGURE-8

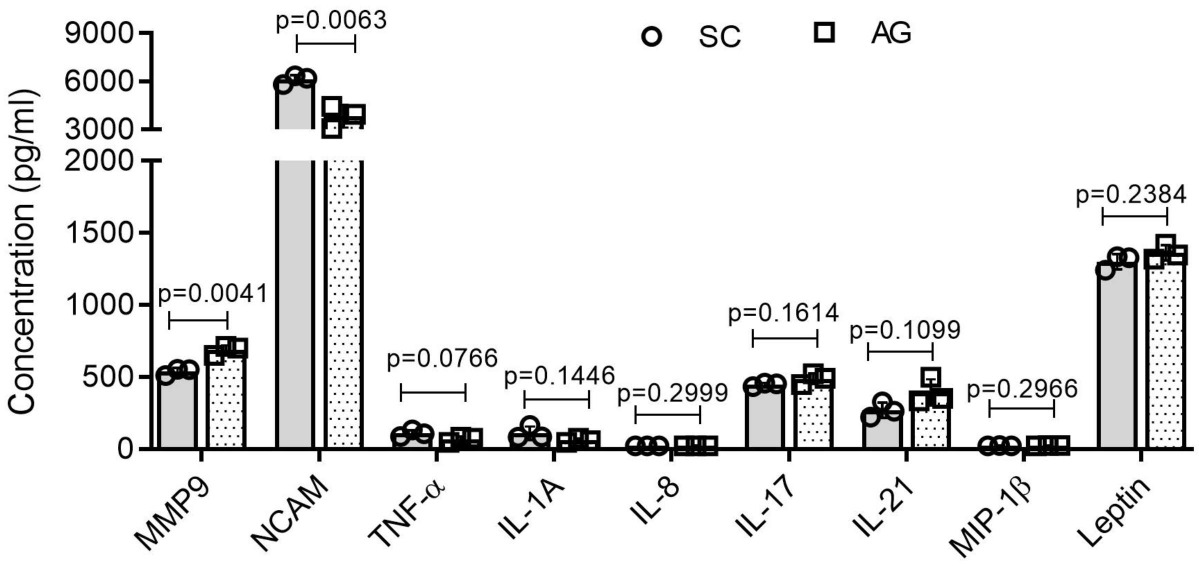

Supplementary Figure-8. Levels of inflammatory cytokines/chemokines in rabbit lungs (n=3) infected with Mtb-SC or AG at 4 weeks. Filtered lung homogenates were used to measure the amount of MMP9, NCAM, TNF-α, IL-1α, IL-8, IL-17, IL-21, MIP-1β and Leptin by ELISA. Values plotted are mean +/- standard error. Data were analyzed by Mann-Whitney U-test.

SUPPLEMENTARY FIGURE-9

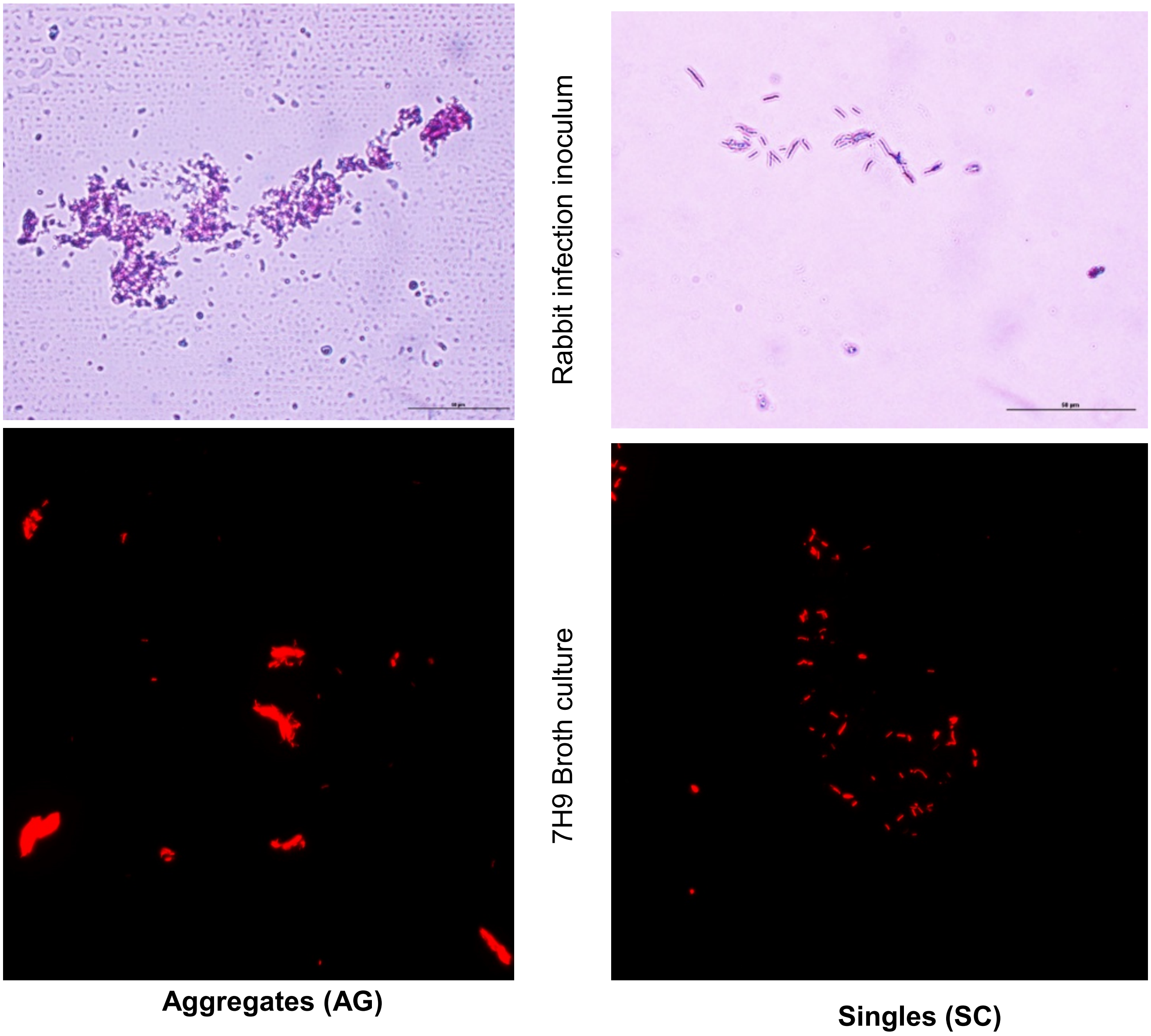

Supplementary Figure-9. Morphology of Mtb in the aerosols of rabbit infection unit (C-H nose-only system) and in 7H9 broth culture. The top panel shows AFB-stained slides of infection inoculum captured at the delivery port of the rabbit infection chamber. Image captured at 630x magnification. Scale bar 10um. The bottom panel shows mCherry-expressing Mtb-H37Rv as aggregates (AG) or singles (SC) grown without or with tween-80, respectively. Image captured at 630x magnification.

SUPPLEMENTARY FIGURE-10.

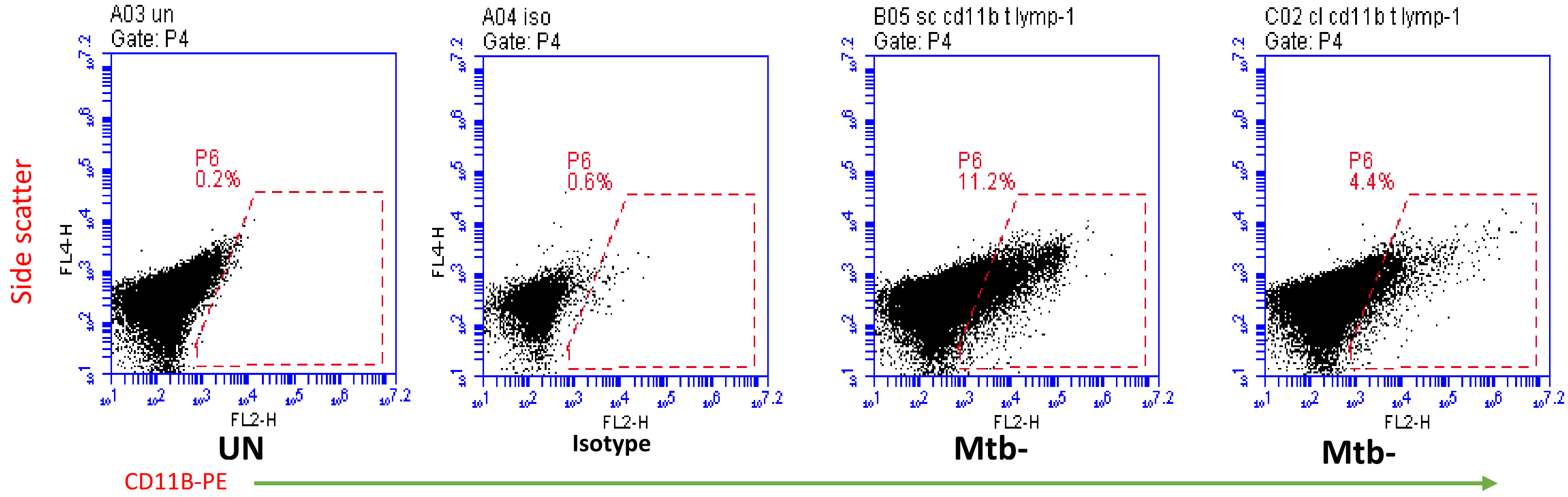

Supplementary Figure-10. Gating strategy used for immune cell profiling by flow cytometry. Figures represent the individual flow cytometry plots of CD11B-PE+ cells in different condition such as unstained (UN), isotype control, Mtb-singles infected or clumps infected respectively.
