# Supplementary material for: Aggregation state of Mycobacterium tuberculosis impacts host immunity and augments pulmonary disease pathology
Source: Commun Biol. 2021 Nov 3;4:1256. doi: 10.1038/s42003-021-02769-9 (PMC8566596; doi:10.1038/s42003-021-02769-9)
Supplement: Supplementary file 9 — Reporting Summary [file 42003_2021_2769_MOESM9_ESM.pdf]

nature portfolio

Corresponding author(s): Selvakumar Subbian

Last updated by author(s): Oct 4, 2021

Reporting Summary

Nature Portfolio wishes to improve the reproducibility of the work that we publish. This form provides structure for consistency and transparency in reporting. For further information on Nature Portfolio policies, see our Editorial Policies and the Editorial Policy Checklist.

Statistics

For all statistical analyses, confirm that the following items are present in the figure legend, table legend, main text, or Methods section.

n/a	Confirmed	
☐	☒	The exact sample size (n) for each experimental group/condition, given as a discrete number and unit of measurement
☐	☒	A statement on whether measurements were taken from distinct samples or whether the same sample was measured repeatedly
☐	☒	The statistical test(s) used AND whether they are one- or two-sided<br>*Only common tests should be described solely by name; describe more complex techniques in the Methods section.*
☒	☐	A description of all covariates tested
☐	☒	A description of any assumptions or corrections, such as tests of normality and adjustment for multiple comparisons
☐	☒	A full description of the statistical parameters including central tendency (e.g. means) or other basic estimates (e.g. regression coefficient) AND variation (e.g. standard deviation) or associated estimates of uncertainty (e.g. confidence intervals)
☐	☒	For null hypothesis testing, the test statistic (e.g. F, t, r) with confidence intervals, effect sizes, degrees of freedom and P value noted<br>*Give P values as exact values whenever suitable.*
☒	☐	For Bayesian analysis, information on the choice of priors and Markov chain Monte Carlo settings
☒	☐	For hierarchical and complex designs, identification of the appropriate level for tests and full reporting of outcomes
☐	☒	Estimates of effect sizes (e.g. Cohen's d, Pearson's r), indicating how they were calculated

Our web collection on statistics for biologists contains articles on many of the points above.

Software and code

Policy information about availability of computer code

Data collection

BD Accurri C6
Nikon Microphot DXM 1200C microscope
Zeiss 200M fluorescent microscope
PathScan Enabler
Promega GloMax plate reader
EVOS FL microscope
Agilent ARIA qPCR unit
Agilent Bioanalyzer
TrimGalore software 16
Lexogen STAR aligner

Data analysis

BD Accurri Software ver. C6 Plus
Nikon NIS Viewer Elements (2016)
Image J (2020)
SigmaScan Pro software
Zeiss Metamorph ver 7.7.9.0
Promega GloMax Software ver 3.2.3
EVOS FL Auto 2 Software ver 2.0.2094.0
Agilent ARIA Software
Qiagen IPA Software (2020)

Partek Genomics Suite ver 7.0
GraphPad Prism ver 9.0
Microsoft Excel 2007

For manuscripts utilizing custom algorithms or software that are central to the research but not yet described in published literature, software must be made available to editors and reviewers. We strongly encourage code deposition in a community repository (e.g. GitHub). See the Nature Portfolio guidelines for submitting code & software for further information.

Data

Policy information about availability of data

All manuscripts must include a data availability statement. This statement should provide the following information, where applicable:

- Accession codes, unique identifiers, or web links for publicly available datasets
- A description of any restrictions on data availability
- For clinical datasets or third party data, please ensure that the statement adheres to our policy

All the data that support the findings of this study are available within the current manuscript and associated supplement. Any additional data can be requested from the corresponding authors upon reasonable request.

Field-specific reporting

Please select the one below that is the best fit for your research. If you are not sure, read the appropriate sections before making your selection.

☒ Life sciences ☐ Behavioural & social sciences ☐ Ecological, evolutionary & environmental sciences

For a reference copy of the document with all sections, see nature.com/documents/nr-reporting-summary-flat.pdf

Life sciences study design

All studies must disclose on these points even when the disclosure is negative.

Sample size	Since this was a model establishment study, and due to non existent data available on rabbits, the sample sizes were determined based on our prior experience on infectious disease research in this model.
Data exclusions	No data were excluded.
Replication	All attempts at replication were successful and included in data analysis<br>Lung bacterial load: n=6-11 per group per timepoint<br>Immunofluorescent microscopy: 24-30 fields per lung (n=4 rabbits per group per time point.<br>Mtb CEQ assay:n=3 rabbits per group per time point and repeated twice.<br>T ell assay:n=3 rabbits per group per timepoint<br>Flow cytometry: 1 sample per subject at each timepoint, n=3 rabbits per group per timepoint<br>Morphometry: 24-30 fields per animal; n=4 rabbits per group per time point<br>Pathology: n=6 rabbits per group<br>Multiplex Cytokine Analysis: Duplicate<br>qPCR: n=3-4 rabbits per group per time point and repeated at least twice
Randomization	Individual animals were randomly assigned to different groups.
Blinding	Support staff, technicians and investigators only involved in data collection were blinded to study groups. Due to involvement of some investigators in animal monitoring and managing the experiments throughout the study, complete blinding was not possible. Few of these investigators were involved in analysis and double-blinded data analysis was not achieved.

Reporting for specific materials, systems and methods

We require information from authors about some types of materials, experimental systems and methods used in many studies. Here, indicate whether each material, system or method listed is relevant to your study. If you are not sure if a list item applies to your research, read the appropriate section before selecting a response.

Materials & experimental systems

n/a	Involved in the study	
☐	☒	Antibodies
☒	☐	Eukaryotic cell lines
☒	☐	Palaeontology and archaeology
☐	☒	Animals and other organisms
☒	☐	Human research participants
☒	☐	Clinical data
☒	☐	Dual use research of concern

Methods

n/a	Involved in the study	
☒	☐	ChIP-seq
☐	☒	Flow cytometry
☒	☐	MRI-based neuroimaging

Antibodies

Antibodies used	A description of all antibodies used is provided in the Methods section
Validation	A description of antibodies specificity and supplier information are provided in the Methods section

Animals and other organisms

Policy information about studies involving animals; ARRIVE guidelines recommended for reporting animal research

Laboratory animals	Details rabbits used in this study is provided in the Methods section
Wild animals	No wild animals were used in the study.
Field-collected samples	No field collected samples were used in the study.
Ethics oversight	The Institutional Biosafety Committee and the Institutional Animal Care and Use Committee (IACUC) of Rutgers University approved all procedures involving rabbits. Animals were handled humanely according to the ethical guidelines of the federal Animal Welfare Act (AWA) and the Association for Assessment and Accreditation of Laboratory Animal Care International (AAALAC).

Note that full information on the approval of the study protocol must also be provided in the manuscript.

Flow Cytometry

Plots

Confirm that:

☒ The axis labels state the marker and fluorochrome used (e.g. CD4-FITC).

☒ The axis scales are clearly visible. Include numbers along axes only for bottom left plot of group (a 'group' is an analysis of identical markers).

☒ All plots are contour plots with outliers or pseudocolor plots.

☒ A numerical value for number of cells or percentage (with statistics) is provided.

Methodology

Sample preparation	All details pertaining to sample preparation are included or cited in text and methods section.
Instrument	BD Accurri C6
Software	BD Accurri Software ver. C6 Plus
Cell population abundance	No sorting was done in this study.
Gating strategy	The gating strategy is included as Supplementary Figure 10.

☒ Tick this box to confirm that a figure exemplifying the gating strategy is provided in the Supplementary Information.
